# Active-Offer Nurse-Led PrEP (PrEP-RN) Referrals: Analysis of Uptake Rates and Reasons for Declining

**DOI:** 10.1007/s10461-019-02745-9

**Published:** 2019-11-26

**Authors:** Patrick O’Byrne, Lauren Orser, Marlene Haines

**Affiliations:** 1 School of Nursing, University of Ottawa, 451 Smyth Road, Ottawa, ON K1H 8M5 Canada; 2 Ottawa Public Health, Infectious Diseases and Sexual Health Clinic, Ottawa, Canada

**Keywords:** HIV, Prevention, Nursing, PrEP, Prevention

## Abstract

While pre-exposure prophylaxis (PrEP) is an effective HIV prevention strategy, its uptake is limited. To address barriers, we piloted a nurse-led PrEP clinic in an STI clinic and had public health nurses refer patients during STI follow-up. We recorded the number of PrEP offers and declines and clinic uptake. We conducted a thematic analysis of patients’ responses from nursing notes written at the time patients declined PrEP. From August 6, 2018 to August 5, 2019, nurses offered a PrEP referral to 261 patients who met our criteria; only 47.5% accepted. Qualitative analysis identified four themes: (1) perceptions of risk, (2) lack of interest, (3) inability to manage, and (4) concerns about PrEP. Our patients did not feel sufficiently at-risk for HIV to use PrEP and maintained that PrEP was for a reckless “other”. This analysis sheds light on how assumptions about risk affect PrEP uptake, particularly among those at-risk for HIV.

## Introduction

HIV pre-exposure prophylaxis (PrEP) involves HIV-negative persons taking antiretroviral medications to prevent HIV acquisition. Taken daily with appropriate clinical monitoring, this intervention can prevent HIV acquisition by up to 96% [1]. Access to PrEP, however, has barriers [2, 3]. For example, despite PrEP being approved for use as an HIV prevention medication by Health Canada and the United States Food and Drug Administration, many persons do not have third party insurance plans or have limited yearly coverage, which can make it challenging to cover the $250/month cost for generic formulations (available in Canada) and over $1000 for trade name PrEP (used in the United States) [4].

Another barrier is that some persons with HIV risk factors do not feel sufficiently at-risk for HIV to warrant PrEP [5–8], which relates to perceptions of risk not health systems issues. Studies comparing perceptions of HIV risk against HIV risk scores showed differences in how personal risk-taking was viewed, with many believing their behaviours were “too low-risk” to warrant PrEP [9–16]. While most persons at risk for HIV agreed PrEP was important, many felt this intervention was only necessary for other higher-risk persons, not themselves [17, 18]. Seeing as most HIV transmission involves persons unaware they were exposed to HIV [19, 20], this lack of self-perceived need for PrEP might undermine the potential population-level benefits of PrEP. Indeed, only those who feel they need PrEP seek it. Considering ongoing HIV transmission, this reliance on patient initiation seems inadequate.

To address this situation, we established an active-offer nurse-led PrEP program, entitled PrEP-RN (PrEP-Registered Nurse) [21]. In this project, local public health nurses discussed PrEP with anyone diagnosed with infectious syphilis, rectal gonorrhea or chlamydia, or anyone who had recent sexual contact with someone diagnosed with HIV in the preceding 12 months.^1^ We also had these public health nurses offer PrEP referrals to persons who did not fulfill these criteria but whom the nurses felt were “high risk” based on clinical judgement [21, 22]. Lastly, we offered PrEP referrals to patients who obtained post-exposure prophylaxis (PEP) at our local sexually transmitted infection (STI) clinic [22].

Our goal in running PrEP-RN was two-fold: (1) expand PrEP rollout at the public health level, rather than relying on patients to self-select for this intervention or be identified by healthcare providers; and (2) increase PrEP uptake among persons with objective HIV risk factors. The motivation underlying the latter goal was to refine PrEP delivery among groups with elevated HIV prevalence [23], and not simply label all gay men, for example, as at-risk based on sexual orientation.

As part of this project, we evaluated the number of persons who met our inclusion criteria. We also collected data about the number of patients who accepted PrEP referrals and the reasons why others declined. In this paper, we review the uptake of our active-offer PrEP project and present a thematic analysis of why patients declined PrEP. These findings highlight that the concept of risk is central to using PrEP, both about one’s degree of risk and the risks of PrEP use.

## Project Overview

On August 6th, 2018, we initiated an active-offer nurse-led PrEP referral process through our public health unit in Ottawa, Canada. Locally, gonorrhea, chlamydia, syphilis, and HIV are reportable infections, meaning that positive test reports are referred to health units for surveillance and to ensure appropriate infection management [24]. For surveillance, in 2018, 77 HIV-positive test results were reported to our health unit, for a rate of 7.6/100,000 [25]. For management, public health nurses contact providers to ensure patients are aware of their diagnoses and receive appropriate treatment or referral. These nurses later complete telephone follow-up with patients to provide STI counselling and ensure their sexual partners (“contacts”) receive testing and treatment [24].

As part of our project, we included active-offer PrEP within this public health follow-up. Based on a review of the literature, we identified specific inclusion criteria that correlated with high HIV incidence rates; these included a diagnosis of infectious syphilis, more than one rectal gonorrhea or chlamydia diagnosis in the preceding 12 months, or being named as contact of someone diagnosed with HIV in the last 12 months. Notably, these diagnoses correlated with 10–20% HIV seroconversion rates per annum [22, 26–28]. Our nurses were also encouraged to offer PrEP to anyone they deemed at-risk for HIV based on clinical judgement [18]. While referrals based on clinical judgement might have varied between cases, it allowed nurses to potentially reach other patients who could have been overlooked for a PrEP referral because they did not meet typical risks profiles. As can be seen in Table 1, referrals based on nurses’ judgement were not purely subjective. They were still based on objective indicators and specialized knowledge of HIV related risks; these risks were simply less explicit than our usual criteria.

**Table 1:** Nurses clinical judgment referral criteria

Reason for referral	n/22	%
Chlamydia and/or gonorrhea in alternate site or only one STI diagnosis in the past 12 months	17	77.2
Men who have sex with men	12	54.5
Condomless anal sex	11	50
Multiple/anonymous sexual partners	7	31.5
Partner diagnosed with chlamydia, gonorrhea, or syphilis	5	22.7
Previous history of syphilis (> 12 months ago)	4	18.2
Trans	1	4.5
Illicit drug use	1	4.5

For this project, our nurses fulfilled all required elements of STI follow-up and inquired if patients who met our inclusion criteria were interested in PrEP. The nurses were educated about PrEP, including its indications for use, mechanisms of action, and risks/benefits, including possible secondary effects, follow-up protocols, etc. Nurses were given a script to guide PrEP offers and discussions, and to ensure each patient received the same minimum amount of information, after which patients were asked if they would like a referral. The nurses provided this information to qualifying patients in a 2 to 5-min counselling session, depending on patients’ questions/needs, during STI follow-up. We engaged in monthly check-ins with these nurses to address questions or concerns and to review the referral processes. We obtained funding from the Ontario HIV Treatment Network (OHTN) to provide PrEP medication at subsidized rates (up to free) to patients with financial limitations to ensure cost did not impede PrEP uptake [21, 29, 30]. Patients who met our inclusion criteria could attend one of four clinics in Ottawa; two were community-based infectious disease clinics, one was a hospital-based infectious disease clinic, and one was our nurse-led PrEP clinic (PrEP-RN) [21].

## Methods

### Design

Our overall study design was mixed methods [31], in that we obtained descriptive quantitative data about the participants and overall program operations, and qualitatively reviewed nursing notes. Our mixed design allowed us to situate our understanding of uptake using the quantitative data about rates and the qualitative data about reasons for declining a PrEP referral. We used each of these approaches to inform the other in a true mixed design fashion. Research ethics boards at the University of Ottawa and Ottawa Public Health approved this project.

### Data Collection and Analysis

Our nurses collected data about all patients who fulfilled our inclusion criteria, including age and sex, reason(s) for PrEP referral, if the patient accepted the referral (and to which clinic), and patients’ reasons for declining referral. To ensure an accurate and contemporaneous capture of patients’ reasons for declining PrEP, our nurses recorded verbatim what patients said at the time this occurred on a PrEP referral form; these quotes were later added to a data collection log (Excel spreadsheet). Rather than using pre-determined categories to codify patients’ reasons for declining PrEP, we allowed patients to offer their own rationale after being asked if they wanted a referral. These quotes were one to several sentences long.

To better understand our patients’ decisions for declining PrEP, we applied Guest et al.’s [32] principles of applied thematic analysis to the verbatim text our nurses noted on the declined PrEP referral forms. For this process, first, we reviewed the quotes to gain a global understanding of the material. This involved reading all quotations on all referral forms. Second, we coded these quotations with one or multiple words to capture the patient’s meaning. These codes were, in most instances, the patients’ words, though we used our own words when these were more succinct. Third, we clustered similar codes. At this point, clusters were not named. Fourth, we reviewed the clusters to identify commonalities. Fifth, we generated names for the commonalities in these clusters. These names captured the common sentiment in these clusters and became the themes that we identified and present below. Sixth, we returned to the initial quotations and ensured the thematic names resonated with each quotation. This step involved slight refinement of the thematic names, but not restructuring of the themes.

## Results

### Uptake Data

From August 6, 2018 to August 5, 2019, our nurses identified 340 patients who met our referral criteria. Twenty-three were HIV contacts, 47 had used PEP within the past two years, 36 were diagnosed with more than one rectal gonorrhea or chlamydia infection within the previous year, 96 were diagnosed with infectious syphilis, 47 fulfilled more than one criterion, and 91 were identified based on clinical judgement. Of these 340 patients, 98% (n = 261) were male and 23% (n = 79) were ineligible for PrEP either due to medical reasons (e.g., HIV-positive or already on PrEP) or insurance reasons (e.g., no health insurance). Of the 261 patients who were eligible for a referral, 47.5% (n = 124) accepted and 52.4% (n = 137) declined.

Of note, three of these 261 patients who were eligible for PrEP have since been diagnosed with HIV, yielding a 1.1% positivity rate (n = 3/261) or 1 new HIV infection per 87 patients who met the inclusion criteria of our active-offer PrEP referral project in a jurisdiction with an HIV diagnosis rate of 7.6/100,000 in the same year [26]. One of these diagnoses was a patient who declined a PrEP referral twice within 6 months of being diagnosed with syphilis and rectal chlamydia and gonorrhea; one was a patient who accepted a referral after two syphilis diagnoses as well as rectal and pharyngeal gonorrhea over 3 months but never started PrEP; the third was a patient who was offered PrEP based on our nurses’ clinical judgment but was found to be HIV-positive at baseline, having had negative HIV serology 8 months earlier.

Among the 137 patients who met our inclusion criteria but declined referral, all were male and, on average, 35 years old. Seven of these 137 patients were HIV contacts (of 13 eligible, for a 46.2% acceptance rate), 21 had used PEP (of 42 eligible, for a 50% acceptance rate), 14 had more than one rectal gonorrhea or chlamydia infection (of 20 eligible, for a 30% acceptance rate), 43 were diagnosed with infectious syphilis (of 71 eligible, for 39.4% acceptance rate), 34 were identified based on clinical judgement (of 76 eligible, for a 55.2% acceptance rate), and 18 fulfilled multiple criteria (of 39 eligible, for a 53.8% acceptance rate). The lowest uptake rate was among patients with rectal bacterial STIs or those with multiple risk criteria and the highest was among patients identified as high-risk based on nurses’ clinical judgement. See Table 2.

**Table 2:** Accepted PrEP referrals

Reason for referral	Referrals offered (n = 261)	Total number accepted	Acceptance rate (%)
HIV contact	13	6	46.2
PEP use in past 2 years	42	21	50.0
> 1 rectal chlamydia/gonorrhea infection	20	7	30.0
Infectious syphilis diagnosis	71	28	39.4
Nurses clinical judgement	76	45	55.2
Multiple referral criteria	39	22	53.8

### Reasons for Declining Active-Offer PrEP

Our analysis of the nurses’ notes about declined PrEP referrals identified four main themes. These were as follows: perceptions of risk; lack of interest; inability to manage; and perceptions of PrEP. There were no notable differences in the reasons for declining between those offered due to objective risk criteria and those offered based on nurses’ clinical judgement. Of note, 8% (n = 11/137) of patients who declined a PrEP referral could not be classified because no information was collected or no explanation was provided for their decision. We thus had detailed explanations about why 92% of our cohort declined PrEP.

#### Finding 1: Perceptions of Risk

The most common reason for declining PrEP related to patients’ perceptions of risk. This group accounted for 42.3% (n = 58/137) of our patients. In these cases, patients felt they were not sufficiently at-risk to warrant PrEP, notwithstanding meeting our inclusion criteria and being explained why PrEP might be beneficial. Our patients’ belief was that PrEP is for persons who do not use condoms with multiple, non-regular, male partners. One patient who used PEP but declined PrEP stated he is too low risk for PrEP because “this was my first anal sex with a man”. Another patient with pharyngeal, rectal, and cervical gonorrhea noted she did not need PrEP because she “rarely has sex with men”. Other participants emphasized the uniqueness of their current situation, and how, while they may seem high-risk, this was atypical. One patient stated, “I’m not at risk. It’s not normally like this”, despite having been diagnosed with syphilis and two rectal chlamydia infections over a 12-month period. Another patient with the same diagnoses stated, “I always use condoms, except for this one time”. An HIV contact likewise stated, “I never have unprotected sex. I think the [lottery] has the same odds as me getting HIV”. Other patients diagnosed with syphilis stated that they did not need PrEP because they were “in a monogamous relationship”, but would consider PrEP in the future if needed. The fact that they had been diagnosed with syphilis while in this “monogamous relationship” did not affect their perceptions of HIV risk and they continued to decline PrEP. It was simply not something they felt they needed.

The point here is that our patients did not deny risks, but stated that their current levels of risk were atypical and that their usual level of risk did not warrant PrEP. These reasons for declining PrEP mostly related to the frequency of sexual contact, infrequent sexual contacts with men, or regular condom use, despite STI acquisition at the anatomic sites where condoms were reportedly used. These discrepancies, nevertheless, did not change perception. It was not, however, that our patients were unconcerned. Aside from the 22 who were identified as HIV contacts, all had self-selected to undergo STI/HIV testing. They thus felt there was some risk; it was simply not enough to warrant PrEP.

#### Finding 2: Lack of Interest

The second finding was that patients were “not interested” in using PrEP when offered, and was the reason given by 26.3% (n = 36/137) of those who declined a referral. These patients were aware of PrEP and knew what it did and how to obtain it, but stated they were either “not interested” or that they “wanted to think about it”. Unique among these refusals was that they included both patients who outright declined PrEP (i.e., were “not interested”) and those who described the sentiments of being in a state of contemplation or precontemplation about PrEP and wanted additional time to consider their options. In the latter cases, we provided relevant information about PrEP and how to obtain it in the future, if the patient wanted to do so. Of these cases, four returned for PrEP and were referred. Interestingly, some of these refusals may have related to the timing of when PrEP was offered. For example, among the 27 patients who obtained PEP during our study period and were eligible for PrEP, 74% (n = 20) declined PrEP when initially accessing PEP, but seven more later agreed to a referral at a routine two-week check-in. This change in acceptance may have related to patient priorities at the time when we offered PrEP, thus showing that interventions must align with patient priorities and readiness. Indeed, in our program, offering PrEP when patients sought PEP had a 26% uptake rate, which increased to 52% (n = 14/27) when PrEP was re-offered  two weeks later, highlighting that interest in and uptake of PrEP may be temporally mediated.

#### Finding 3: Inability to Manage

The third finding related to life context, and was raised by 13.8% (n = 19/137) of our patients. These patients neither opposed PrEP nor had negative perceptions of it. They simply stated that other items in their life precluded its use. Some of such reasons were mental health, with one patient who was diagnosed with syphilis three times stating he was “not feeling mentally capable at this time”. Another patient with syphilis and urethral chlamydia twice, who reported engaging in condomless receptive anal sex with multiple male partners, stated he “needed to get things in my life in order first”. Others stated they were travelling and could not attend a referral. Interestingly, five participants declined PrEP due to cost, despite our nurses explaining that subsidized medication was available. The explanation of cost may have been a convenient excuse for patients who did not want to use PrEP otherwise. Nevertheless, this set of findings highlighted that, while patients may benefit from PrEP, it may not be a priority for them when healthcare providers raise it. Again, timing and fit for the patient are likely relevant factors for PrEP initiation.

#### Finding 4: Perceptions of PrEP

The final common reason why our patients declined PrEP related to perceptions of this intervention. Among those who declined PrEP for this reason, which accounted for 9.5% (n = 13/137) of all patients who declined PrEP, these beliefs were mostly negative. Indeed, among this group, the most common reason for declining PrEP related to beliefs that PrEP was “harmful” and “dangerous”, which encompassed patients’ negative perceptions about the side effects and harms of long-term PrEP use. One participant, who was diagnosed with syphilis and pharyngeal gonorrhea, was an HIV contact, and who reported recreational drug use summarized this belief when he stated, “I don’t want to put that shit in my body; it’s poison”. Another participant with multiple bacterial STIs declined PrEP because he previously used PEP, which “made me sick”. He thus declined a PrEP referral, assuming he would experience side effects similar to when he used PEP.

Another important aspect of this finding was that it included our participants’ perceptions that PrEP use indicates that a person engages in so-called “high risk” behaviour. One participant who was diagnosed with syphilis summarized this as follows: “I don’t want my employer to see that I use PrEP”. The patient was concerned his employer would see these details and deem his PrEP use as an indication or admission of HIV risk practices. This point differs from finding 1, in that, above, the participants declined PrEP due to personal perceptions of their levels of risk (that they were not at-risk), while, for finding 4, participants declined PrEP to prevent others from perceiving them as “risky persons”. These patients did not deny they might be at-risk for HIV; they simply did not want outward indications of this risk, and considered PrEP to be such an indication.

As a whole, this section suggests persistent beliefs about HIV prevention services as an indication of risk-taking behaviour. It also highlights ongoing beliefs about the harmfulness of HIV medications, generally, and the stigma that surrounds these interventions.

## Discussion

In this paper, we presented the findings of our active-offer nurse-led PrEP program, which ran through our public health unit and STI clinic and targeted patients either with objective risk factors for HIV acquisition or who had been identified for PrEP based on nurses’ clinical judgement. We identified that 47.5% of our 261 eligible patients accepted a PrEP referral, which is lower than most published studies [33–35]. Our methodology may account for this outcome. In contrast to previous studies, we recruited through public health nurses completing routine STI case management, meaning that most of our patients were offered PrEP by phone from someone they had never met at the time of an STI diagnosis or potential HIV exposure. Timing and delivery of this offer may have affected uptake. Our finding about life context and PrEP uptake among patients who used PEP, moreover, reinforces the importance of ensuring that PrEP is offered when patients are open to it.

In addition, by using objective risk criteria and nurses’ judgement, patients may be offered PrEP multiple times (e.g., with clinical visits, subsequent STI diagnoses, etc.), which allows patients time to consider referral options and/or obtain additional information about PrEP. This point of repeat offers has proven successful in other studies of PrEP uptake by reducing barriers and supporting patients in moving from a pre-contemplative state to a contemplative or preparative stage [36]. This could be useful for patients in finding 3, who were unable to manage a PrEP referral at the initial point of offer, but might be more prepared at a subsequent visit or with a subsequent offer from a public health nurse. More research is needed to determine optimal timing and number of times to offer PrEP to increase uptake.

Furthermore, while our acceptance rate was below 50%, we nevertheless feel that our program was successful because the 124 patients who accepted a referral initiated PrEP when they otherwise might not have. These 124 patients did not seek PrEP, but agreed to use it after nurses raised the idea. As the number needed to treat to avert one HIV infection ranges from 20 to 40 in the PrEP literature [37–39], this uptake could mean that 3–6 HIV cases were averted during our 1-year study period. In our local context of a 7.6/100,000 HIV diagnosis rate and our study context of 1 HIV diagnosis per 87 patients, we might have averted 1.4 cases during our study.

Beside the purely biologic benefits of HIV prevention, PrEP counselling by public health nurses to persons at higher-risk for HIV has the ancillary benefits of social awareness and public education. Despite the fact that over half of our cohort declined a PrEP referral, all patients received the same basic points about PrEP, which could be shared within their social networks to increase reach about PrEP use and availability to others. This point adds to the utility of having public health nurses, as non-prescribers, in providing HIV prevention counselling, as it helps to develop patient capacity at an individual and group level.

Based on our thematic analysis of patients’ statements, the most common reason for declining a referral was a lack of perceiving oneself as sufficiently at risk for HIV to need PrEP. Our patients viewed the risk for HIV acquisition as a continuum, with PrEP reserved for “others” at the high-end of the spectrum. It was not that our patients denied being at any risk: indeed, they were diagnosed with STIs or accessed HIV testing and PEP based on decisions to seek care. They simply did not think that their level of risk warranted a prevention intervention involving daily medication and clinical/serologic follow-up every 3 months [1]. In other words, our patients’ decision to decline PrEP was, in part, about the balance between the risks of HIV acquisition versus the risks related to, and the investment involved with, obtaining and taking a daily medication. It is possible that more patients might have agreed to PrEP were the pill burden or clinical monitoring less intense. This also applied to the patients who felt that PrEP was potentially harmful: for them, the risks of harm from PrEP exceeded the risk of HIV acquisition and was beyond what they felt they needed for their perceived level of risk.

Our participants, however, did not engage in risk assessments in a purely epidemiologic sense. For them, risk was not just the probability of an outcome occurring; ideas about the *type* of person who needs PrEP pervaded their assessments, with PrEP being reserved for a reckless and dangerous “other”. These *others* were often viewed as persons on the social fringe who lacked self-discipline and were careless with their bodies—i.e., those “likely” to become HIV-positive [40]. Our patients thus not only declined PrEP on the basis of perceiving themselves as being low-risk for HIV acquisition, but also because they did not want to be categorized, by nurses and others, as this stigmatized high-risk *other*. Explained differently, nearly half of our participants declined PrEP because they had preconceived ideas of the “type of person” who required PrEP (and did not see themselves as such a person) or because they did not want to be viewed as part of a reckless *othered* group of, mostly gay men, who engage in practices that might transmit HIV. This point, in short, related to stigma, which refers to the possession of an attribute that serves to discredit an individual and which is the basis for social exclusion [41]. Individuals who possess certain personal and/or behavioural characteristics, such as sex with same sex partners, being in a sero-discordant relationships, or PrEP use, are considered to be outside of what is socially desirable, or conventionally “normal”, are subconsciously labelled as “abnormal”, or in this case, “risky” [41]. Stigma seemed to play a strong role in the reasons why our participants declined PrEP.

Lupton’s [40] work helps explain the relationship between stigma and risk. Building on Douglas and Castel, Lupton posited that risk connotes danger and dangerousness, with risk increasing as items become increasingly socially unacceptable [40]. Central to the conception of risk is the idea that *intransgressible* boundaries were breached by persons engaging in deviant acts of condomless sex with multiple male partners [40, 42]. By extension, people who possess these so-called risky attributes are considered dangerous and dirty, and become the stigmatized *other* against whom protection is required [35]. These are the people who crossed a boundary that is prohibited socially. The othering attached to risk, however, does not end there; it includes those deemed at high risk for the negative outcome of interest, in this case HIV [40]. That is, while persons living with HIV are stigmatized based on their serostatus, so too are those who are high-risk for acquiring the infection. This extension of othering to those who are at high-risk for HIV relates to the fact that contemporary mainstream perceptions hold that people should naturally want to and actively undertake actions to avoid, mitigate, or reduce the risks of HIV acquisition [40, 43]. In other words, at-risk individuals have a responsibility to prevent unwanted outcomes (i.e., HIV-positivity), with a failure to do so constituting irrational and reckless behaviour. This group, therefore, is stigmatized due to their potential to become HIV-positive.

What we see with PrEP, and especially our active-offer PrEP referral program, is the construction of the “at-risk person and body” [40]. We used STI diagnoses, PEP use, and clinical judgement to identify the at-risk person and actively offer them an additional prevention strategy. Seen in this way, our criteria and process fulfill what Lupton [40] suggested: a systematic way to identify and regulate deviant bodies under the guise of helping them mitigate risks that are deemed inherently negative. As might be expected, nearly half our patients rejected this process, refusing to be seen or to see themselves as at-risk, particularly those who believed they were in monogamous relationships, or who “never” have anal sex with other men. Lupton’s work [40] can be used to explain this finding as follows: in declining PrEP, the person, on the one hand, rejects a stigmatizing label (of being the at-risk other), while, on the other hand, does not acquire the responsibilities that are socially associated with being at-risk. In other words, in rejecting PrEP, patients protect their self-image and limit their responsibility, enabling them to continue status quo [40]. Lupton’s work makes rational sense of our patients’ rejection of PrEP, which from mainstream societal and healthcare perspectives is an irrational action, especially considering the three patients in our group who became HIV-positive within months of being offered PrEP.

This point is important. It emphasizes that nurses and other healthcare providers and workers need to recognize that, while HIV acquisition does have antecedents (our so-called objective risk factors and the clinical risk factors our nurses used), the understanding of risk in everyday life is subjective and culturally bound. As such, nurses should not attempt to correct or impose dominant ideologies about risk on patients, but rather, should provide care that aligns with patients’ views about risk and concurrently help them minimize the possibilities of HIV acquisition.

As a final point, while our indications for a PrEP referral did not focus on sexual orientation or practices, we still primarily offered PrEP to men who have sex with men, maintaining the perception of and focus on this one group in public health HIV prevention work. Indeed, over half of our referrals involving clinical judgement involved men who have sex with men, albeit likely because our referral criteria were based on infections that primarily affect this group: rectal bacterial STIs, infectious syphilis, and HIV. Although this emphasis on men who have sex with men is important, as HIV continues to unequally affect this group, our approach nonetheless contributes to a long history of medical interventions aimed at regulating the gay male body. This fits with Lupton’s assertions about risk [40], in that, just like our patients, our allegedly epidemiologic and empirical risk assessments served to identify persons who fall outside the socially accepted standards of monogamous coupled heterosexual relationships. We did not necessarily address the social inequities that render these men more affected by HIV; we simply identified them and offered a prevention tool.

Despite this criticism, we think active-offer PrEP should continue, but with efforts to better understand the sociological implications of this approach. The next phase of our research will ths involve qualitative interviews with patients who declined or accepted PrEP referrals to better understand their views on PrEP and risk. The knowledge gained from these interviews could help modify our referral process, including determining the ideal time and settings to offer PrEP (e.g., over the phone versus in person) and to facilitate broader access to PrEP among populations with elevated HIV prevalence, including persons of African, Black, or Caribbean ethnicity, persons who are Indigenous, and persons who are transgender.

## Limitations

Some limitations exist with the use of PrEP referrals based on objective risk assessments and clinical judgement. While empirically based, our risk criteria depended on patients (1) accessing STI testing, and (2) having a positive diagnosis, which could limit referrals to men who have sex with men, who more often engage in routine STI screening or who may see healthcare providers who do more comprehensive examinations (e.g., rectal or pharyngeal swabs). Thus, the ability to offer PrEP to other groups with elevated HIV prevalence [23], such as persons who use injection drugs, African, Caribbean, or Black persons, Indigenous groups, and persons who are transgender, might be restricted by our referral criteria. Moreover, while the clinical judgement category incorporated known risk factors for HIV acquisition, some nurses also considered patients’ sexual orientation and condomless sex as components of risk, which could perpetuate stereotypical views of “risky behaviour” among gay men. One way to reduce unintentional targeting of PrEP at gay men could be to provide PrEP counselling to all patients during every clinical visit and/or public health follow-up. However, consideration must be made for the additional time this might take and the potential to perpetuate HIV/AIDS related anxieties among lower-risk groups.

## Conclusion

In this paper, we presented the findings of our active-offer PrEP referral project, in which we had nurses offer patients PrEP as part of routine public health STI case management. While half our patients declined a referral, the other half agreed, which we took as a sign of project success because we linked these patients with PrEP when they otherwise would not have. Our thematic analysis of the nursing notes about why patients declined PrEP, moreover, highlighted that many patients did not view themselves as being sufficiently at-risk for HIV to warrant PrEP, notwithstanding risk factors that correlated with elevated HIV incidence rates in the published literature and our study. This finding highlights the need for more research about people’s perceptions of HIV risk and PrEP, and how, when, and why these feel this intervention would be necessary. Considering ongoing HIV transmission, better tailoring PrEP to persons who could benefit it might yield tangible HIV prevention outcomes at the individual and population levels.

## Footnotes

1 While most persons achieve an undetectable viral load within 6 months of treatment, we used 12 months because it encompasses not only the period of medication initiation to viral load suppression (often 6 months), but also the time from diagnosis to referral to care to medication initiation (which can range from days to another few months).
